# MicroRNA‐130a inhibits proliferation of vascular smooth muscle cells by suppressing autophagy via ATG2B

**DOI:** 10.1111/jcmm.16305

**Published:** 2021-02-21

**Authors:** Liang Zheng, Zhecun Wang, Zilun Li, Mian Wang, Wenjian Wang, Guangqi Chang

**Affiliations:** ^1^ Laboratory of General Surgery Division of Vascular Surgery National‐Guangdong Joint Engineering Laboratory for Diagnosis and Treatment of Vascular Diseases The First Affiliated Hospital Sun Yat‐sen University Guangzhou China

**Keywords:** arteriosclerosis obliterans, ATG2B, autophagy, microRNA‐130a, vascular smooth muscle cells

## Abstract

Numerous microRNAs participate in regulating the pathological process of atherosclerosis. We have found miR‐130a is one of the most significantly down‐regulated microRNAs in arteriosclerosis obliterans. Our research explored the function of miR‐130a in regulating proliferation by controlling autophagy in arteriosclerosis obliterans development. A Gene Ontology (GO) enrichment analysis of miR‐130a target genes indicated a correlation between miR‐130a and cell proliferation. Thus, cell cycle, CCK‐8 assays and Western blot analysis were performed, and the results indicated that miR‐130a overexpression in vascular smooth muscle cells (VSMCs) significantly attenuated cell proliferation, which was validated by an in vivo *assay* in a rat model. Moreover, autophagy is thought to be involved in the regulation of proliferation. As our results indicated, miR‐130a could inhibit autophagy, and ATG2B was predicted to be a target of miR‐130a. The autophagy inhibition effect of miR‐130a overexpression was consistent with the effect of ATG2B knockdown. The results that ATG2B plasmids and miR‐130a mimics were cotransfected in VSMCs further confirmed our conclusion. In addition, by using immunohistochemistry, the positive results of LC3 II/I and ATG2B in the rat model and artery vascular tissues from the patient were in accordance with in vitro data. In conclusion, our data demonstrate that miR‐130a inhibits VSMCs proliferation via ATG2B, which indicates that miR‐130a could be a potential therapeutic target that regulates autophagy in atherosclerosis obliterans.

## 1 INTRODUCTION

A large number of arteriosclerosis obliterans (ASO) cases occur in the South‐East Asia and Western Pacific regions.^1^ ASO affects mainly the small‐ and medium‐sized arteries of the lower limbs and is characterized by lipid and inflammatory cell accumulation within the intima of large arteries.^2^ Endovascular surgery has a good curative effect on ASO; however, the vascular restenosis that results from neointimal hyperplasia remains a problem, leading to the failure of revascularization procedures.^3^, ^4^ Vascular stenosis is associated with abnormal proliferation and migration of vascular smooth muscle cells (VSMCs), which are involved in ASO development.^5^, ^6^ Thus, a sufficient understanding of VSMC proliferation can help to improve ASO therapy.

MicroRNAs (miRs) can serve as significant regulators of the pathophysiological process of ASO, especially cell proliferation and migration, which is closely related to neointimal hyperplasia.^7^, ^8^ Our previous studies showed that miRs, such as miR‐24‐3p, miR‐21 and miR‐22‐3p, regulated VSMC proliferation and migration through modulating direct targets,^9^, ^10^, ^11^ and the DNA methylation of miR‐1298 affected the functions of VSMCs.^12^ Furthermore, Li et al^13^ found that miR‐130a, miR‐27b and miR‐210 could be novel biomarkers for early‐stage ASO. And miR‐145 inhibits the proliferation and migration of VSMCs.^6^ These studies indicate the important role of miRs in the development of ASO.

Autophagy is an important biological process that can regulate cytogenesis and cell survival and protect against cell dysfunction.^14^ It is well established that the autophagy inhibitor 3‐MA can prevent post‐treatment restenosis by inhibiting autophagy and reducing VSMC proliferation, suggesting that autophagy could influence cell proliferation in ASO.^15^ These findings suggest that autophagy participates in the development of ASO and that cell proliferation may be involved in the regulatory process.^16^, ^17^, ^18^, ^19^

Our previous study found that miR‐130a expression was significantly lower in ASO arterial tissues than in control arterial tissues.^9^ Furthermore, Li et al reported that miR‐130a was a candidate biomarker of ASO.^13^ Wu et al^20^ revealed that miR‐130a mediates VSMC proliferation in hypertension. In addition, several studies demonstrated that miR‐130a could regulate autophagy.^21^, ^22^, ^23^, ^24^ Thus, our study aimed to explore the role of miR‐130a in regulating cell autophagy and proliferation in ASO.

## 2 MATERIALS AND METHODS

### 2.1 Cell culture

Primary VSMCs were obtained from the femoral artery of healthy organ donors (n = 3: male, 18 years; male, 18 years; male, 20 years) with the consent of donor and approval of the Ethics Committee of the First Affiliated Hospital of Sun Yat‐sen University. VSMCs were cultured in DMEM (Gibco, Grand Island, NY) supplemented with 20% FBS (Gibco) and 1% penicillin/streptomycin (Gibco) and maintained at 37°C in a humidified incubator containing 5% CO_2_. HEK293T/17 (ATCC, Rockefeller, MD) was cultured in DMEM supplemented with 10% FBS (Gibco) and 1% penicillin/streptomycin (Gibco) and maintained at 37°C in a humidified incubator containing 5% CO_2_.

### 2.2 Cell transfection

Vascular smooth muscle cells were plated in 6‐well plates and allowed to reach 50%–70% confluence at the time of transfection. miR‐130a expression was induced by transfecting target cells with miRNA mimics or miRNA mimic‐negative control (RiboBio, Shanghai, China) using Lipofectamine RNAiMAX (Invitrogen, Carlsbad, CA). VSMCs were also transfected with small interfering RNA (siRNA; RiboBio) to knock down ATG2B using Lipofectamine RNAiMAX (Invitrogen). The plasmids pCDNA3.1(+)‐ATG2B were purchased from Generay (Shanghai, China), and the plasmid transfection was performed by Lipofectamine 3000 (Invitrogen). The small interfering RNAs (siRNAs) targeting ATG2B are listed as follows: siATG2B‐1: sense 5′‐GCAGTAGCTTTCTTTACTT‐3′; siATG2B‐2: sense 5′‐GCTGTCTGTTGCCGTTAAA‐3′; miR‐130a mimics: sense 5′‐CAGUGCAAUGUUAAAAGGGCAU‐3′, antisense 5′‐AUGCCCUUUUAACAUUGCACUG‐3′; and miR‐130a inhibitor: 5′‐AUGCCCUUUUAACAUUGCACUG‐3′.

### 2.3 Determination of cell viability

Cell viability was determined using a Cell Counting Kit‐8 (CCK‐8) (Dojindo, Kumamoto, Japan) assay according to the manufacturer's instructions. Cells were grown in 96‐well plates and incubated with CCK‐8 reagent for 3 hours at 37°C. Absorbance was measured at 450 nm.

### 2.4 Flow cytometry

Flow cytometry was used to determine the cell cycle regulation of VSMCs transfected with miR‐130a mimics. VSMCs were fixed with cold 70% ethanol overnight at 4°C. After the ethanol was removed, the cell pellet was resuspended in FACS buffer (containing 50 μg/mL propidium iodide and 100 μg/mL RNase A) and incubated for 1 hour at room temperature. The samples were analysed using a CytoFLEX flow cytometer (Beckman Coulter, Brea, CA).

### 2.5 Immunofluorescence assay

Vascular smooth muscle cells were seeded on 13‐mm‐diameter cover slides. Then, the VSMCs were fixed using cold paraformaldehyde (4%) at 4°C for 20 minutes, followed by permeabilization with cold methanol (−20°C) for 5 minutes. Subsequently, the cells were incubated with a mouse anti‐smooth‐muscle (SM) α‐actin antibody (Sigma‐Aldrich, St. Louis, MO), calponin antibody (Abcam, UK) and MYH11 antibody (Abcam) at 4°C overnight. Next, the cells were incubated with an Alexa Fluor^®^ 594 donkey antimouse IgG (H + L) secondary antibody (Invitrogen) at room temperature for 1 hour. Finally, the cells were visualized using a confocal microscope (Zeiss LSM 510; Germany).

### 2.6 Vascular injury model and animal treatment

All protocols were approved by the Institutional Review Board of The First Affiliated Hospital of Sun Yat‐sen University. Sprague Dawley rats (weight: 250‐300 g; Model Animal Research Center of Nanjing University, Nanjing, China) were used.

Rats were maintained at constant humidity (60 ± 5%), temperature (24 ± 1°C) and light cycle (6 am to 6 pm) and were fed a standard diet. Rats were anaesthetized with isoflurane, and the concentration of anaesthesia induction was 3% and anaesthesia maintenance was 2%. Under a dissecting microscope, the left common carotid artery was exposed through a midline cervical incision, and blood flow of the left common, internal, and external carotid arteries was temporarily interrupted by vessel clips. A 2‐Fr Fogarty Catheter (Edwards Lifesciences, Irvine, CA) was introduced through an arteriotomy in the external carotid artery. To achieve carotid artery injury, we inflated the balloon with saline and withdrew it six times from just under the proximal edge of the omohyoid muscle to the carotid bifurcation. After injury, the external carotid artery was permanently ligated with a 6‐0 silk suture. The clips at the common and internal carotid arteries were released to restore the blood flow.

Rats were divided into four groups. Group 1 was uninjured left carotid arteries as sham‐operated control. Group 2 was treated with the lentiviral construct‐empty vector (40 ~ 50 μL, 5 × 10^9^ pfu/mL, pH 7.4) as a control. Group 3 was treated with the LV‐miR‐130a (40‐50 μL, 5 × 10^9^ pfu/mL, pH 7.4). Group 4 was treated with the LV‐miR‐130a inhibitor (40‐50 μL, 5 × 10^9^ pfu/mL, pH 7.4). miR‐130a inhibitor and miR‐130a were cloned into the GV369 vector and were packaged into a lentivirus by Genechem (Genechem, Shanghai, China) (LV‐miR‐130a inhibitor and LV‐miR‐130a). Lentivirus with the empty vector was used as a negative control (oligo control). Six rats were involved in each group.

### 2.7 Transwell assay

For the transwell assay, after the transfection of miR‐130a mimics, siRNA‐ATG2B or pCDNA3.1(+)‐ATG2B, VSMCs were resuspended in serum‐free DMEM (5 × 10^5^ cells/mL), and 200 µL of the solution was added to the upper transwell chamber (Costar, NY). The lower chamber was filled with DMEM (Gibco) supplemented with 10% FBS (Gibco). After 24 hours of incubation, the cells that migrated to the lower surface of the chamber membrane were fixed with paraformaldehyde (4%) and subsequently stained with 0.1% crystal violet in 20% methanol, whereas the cells that remained on the upper surface of the membrane were removed with a cotton swab. After 3 washes with PBS, the migrated VSMCs were counted under an inverted microscope (Leica DMI4000B).

### 2.8 Wound closure assay

For the wound closure assay, after miR‐130a mimic or siRNA‐ATG2B transfection, VSMCs were seeded into 12‐well plates (12 000 cells/well). A single scratch wound was generated using a sterilized 200‐μL disposable pipette tip. Scratch wounds were visualized using an inverted microscope (ZEISS), and the widths of the scratch wounds were measured using Image‐Pro Plus 6.0 software. Wound closure (%) indicates the percentage of wound closure with the initial scratch width set as 100%.

### 2.9 Reverse transcription‐quantitative polymerase chain reaction (RT‐qPCR)

RNA extraction was performed using TRIzol (Invitrogen) according to the manufacturer's protocol. Then, 1 μg of RNA was reverse‐transcribed into cDNA. Next, the threshold cycle (Ct) value was determined, and the relative miR‐130a level was calculated based on the Ct values and normalized to the U6 level in each sample. PCR was performed with the following primers: miR‐130a‐3p, forward 5′‐GGCAATGTTAAAAGGGCATAAA‐3′; U6, forward 5′‐GGAACGATACAGAGAAGATTAGC‐3′ and reverse 5′‐TGGAACGCTTCACGAATTTGCG‐3′.

### 2.10 Western blot analysis

Protein samples were obtained from the lysates of cultured cells, and the protein concentrations were determined. The samples were separated by SDS‐PAGE, transferred to a PVDF membrane and blocked with TBST (TBS containing 0.05% Tween‐20) and 5% non‐fat milk powder for 2 hours. The membranes were then incubated with the following specific primary monoclonal antibodies overnight at 4℃: SQSTM1/P62, cyclin D1, Beclin1, ATG7, p16 INK4A, GAPDH (Cell Signaling Technology, MA), LC3 II/I and ATG2B (Sigma, St. Louis, MO). After 5 washes with TBST, the membranes were incubated with an IgG‐hydrogen peroxide (HRP) secondary antibody (Cell Signaling Technology) for 1 hour at room temperature. The protein samples were visualized using a chemiluminescence method.

### 2.11 Immunohistochemistry staining

Formalin‐fixed and paraffin‐embedded tissues were sectioned at 4 μm. Tissue slides were deparaffinized in xylene and rehydrated in a graded series of ethanol. Antigen retrieval was performed by microwave heating. Non‐specific antigens were blocked with 1.5% normal goat serum. Primary antibody for ATG2B and LC3 II/I (1:50 dilution) was incubated on the slides overnight at 4°C. Then, the slides were incubated with secondary antibody and sections were then stained with diaminobenzidine and counterstained with haematoxylin. Further, slides were treated with EnVision™ Detection Systems, Peroxidase/DAB, Rabbit/Mouse (Dako, Santa Clara, CA) according to the manufacturer's instruction, and finally visualized using Eclipse 80i microscope (Nikon). Integrated optical density (IOD) value, which would represent the staining intensity, was calculated using Image‐Pro Plus 6.0 software (Media Cybernetics) on the acquired images. The IOD values of different sections were compared.

### 2.12 Dual‐Luciferase Reporter assay

Fragments of the ATG2B mRNA 3′‐UTRs containing the putative or mutated miRNA‐binding sites for miR‐130a were cloned into the GV272 luciferase reporter vector (GeneChem). The constructs were then cotransfected with miR‐130a mimics or negative control oligos into 293T cells using Lipofectamine 2000 (Invitrogen) according to the manufacturer's instructions. Luciferase activity was measured 48 hours after transfection using the Dual‐Luciferase^®^ Reporter Assay System (Promega, Madison, WI) according to the manufacturer's protocol.

### 2.13 In situ hybridization staining

The localization of miR‐130a was observed by in situ hybridization (ISH). miR‐130a FISH Probe was purchased from Exonbio Inc (Guangzhou China) and labelled with DIG. In situ observation of miR‐130a was performed using 4‐μm sections of samples with a digoxigenin‐labelled oligonucleotide lncRNA‐TR detection probe (Exiqon, Aarhus C, Denmark), as previously described. miR‐130a probe sequence was (5′‐3′): Dig‐5‐ATGCCCTTTTAACATTGCACTG‐3.

### 2.14 Transmission electron microscopy

To measure autophagosomes, cells were seeded and grouped as described above. Cells were harvested by centrifugation at 300 g, and then sectioned and fixed in 4% glutaraldehyde containing 2% paraformaldehyde in 30 mmol/L phosphate buffer, pH 7.4. After dehydration with alcohol, the slides were placed in embedding moulds saturated in propylene oxide and incubated at 60°C for 48 hours. The 70‐nm‐thin sections were prepared and detected by TEM (FEI, Eindhoven, The Netherlands) at 120 kV.

### 2.15 Statistical analysis

Gene Ontology (GO) and Kyoto Encyclopedia of Genes and Genomes (KEGG) pathway enrichment analyses of miR‐130a in humans were conducted with mirPath v.3.0 using the TarBase v7.0 database (ref: DIANA‐miRPath v3.0: deciphering microRNA function with experimental support). miR‐130a target prediction was performed by starBase v3.0 based on the PITA, TargetScan, miRanda and miRmap databases (http://starbase.sysu.edu.cn/). The common predicted targets among the 4 databases were used for GO enrichment analysis by DAVID 6.8 (https://david.ncifcrf.gov/). The results were visualized using the ggplot2 package in R.

All data are expressed as the mean ± standard deviation (SD). Student's t test was used to analyse differences between two groups. A *P*‐value < 0.05 was deemed statistically significant.

## 3 RESULTS

### 3.1 miR‐130a suppressed VSMC proliferation and migration

Firstly, we successfully cultured primary VSMCs with high SM‐α‐actin (red), calponin (green) and MYH11 (red) expression according to the IF assay (Figure S1A). miR‐130a overexpression was also successfully induced through miR‐130a mimic transfection in VSMCs (Figure S1B).

**FIGURE 1:**
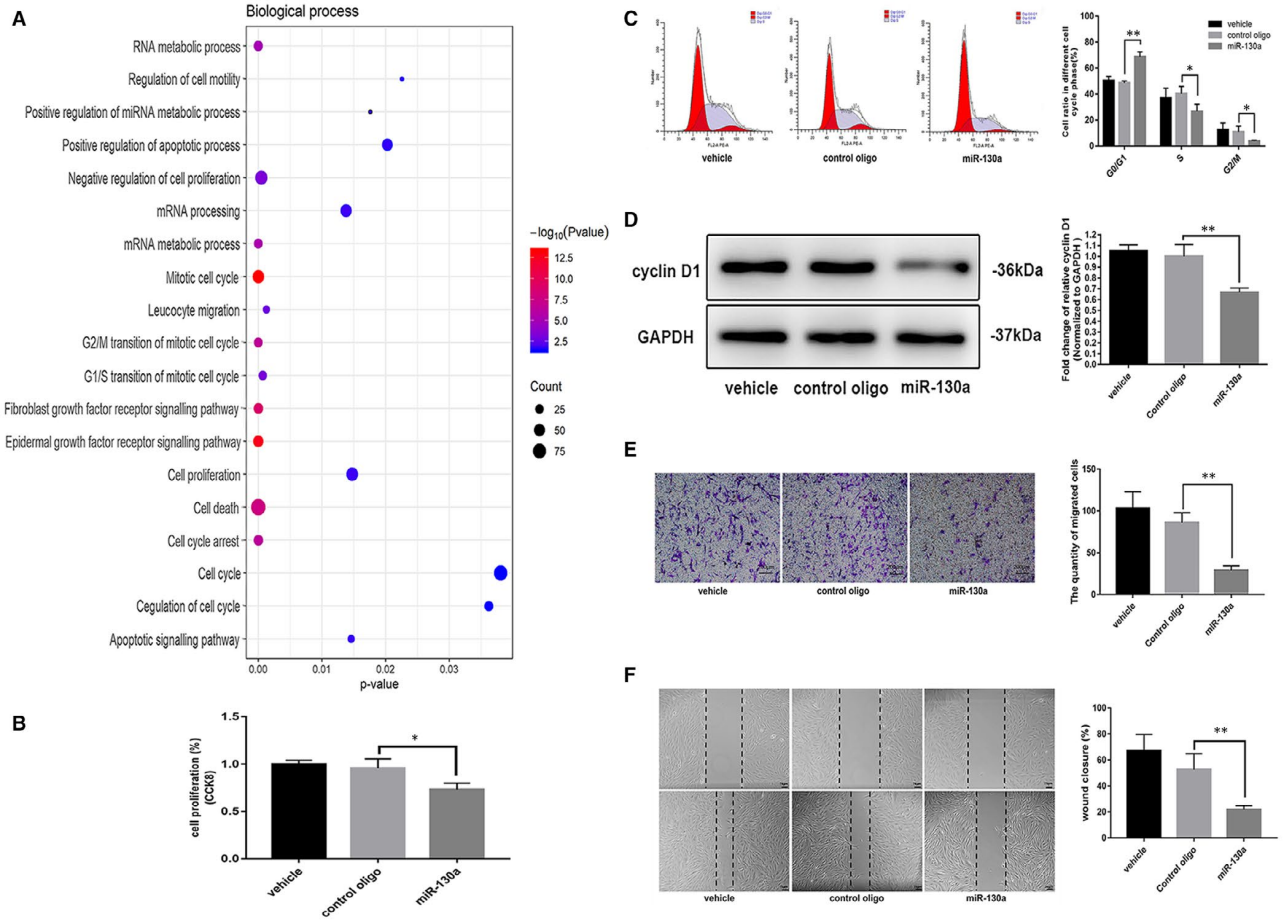
miR‐130a suppressed VSMC migration and proliferation. A, Bubble plot of miR‐130a from the GO enrichment analysis. B, miR‐130a decreased VSMC proliferation as determined by CCK‐8 assay (n = 3 for both). C, A cell cycle analysis using flow cytometry showed that miR‐130a decreased VSMC progression into the S phase (n = 3 for both). D, The levels of cyclin D1 and host cell GAPDH were measured by Western blotting (n = 3 for both). E, miR‐130a decreased VSMC migration as determined by transwell assay (n = 3 for both). Scale bar = 200 μm. F, miR‐130a decreased VSMC migration as determined by wound closure assay (n = 3 for both). Scale bar = 75 μm. *P < 0.05; **P < 0.005; ***P < 0.001

Vascular smooth muscle cells proliferation leads to the growth of plaques in the intima and media layers of arteries, resulting in partial or total obstruction of the vascular lumen,^5^ and our GO enrichment analysis of miR‐130a target genes indicated that miR‐130a was obviously related to cell proliferation regulation (Figure 1A). Thus, we first explored whether miR‐130a could regulate VSMC proliferation. A CCK‐8 assay demonstrated that cell proliferation was significantly inhibited after miR‐130a overexpression in VSMCs (Figure 1B).

To further explore the mechanism by which VSMC proliferation was inhibited after miR‐130a overexpression, a flow cytometry analysis was performed to detect changes in the cell cycle. The results indicated that miR‐130a induced significant G0/G1 phase cell cycle arrest, and accordingly, the ratio of cells in S phase was significantly lower in miR‐130–overexpressing VSMCs than in negative control cells (Figure 1C). These results were validated according to the expression of cyclin D1.^25^ Our data indicated that cyclin D1 was decreased dramatically in miR‐130a mimic VSMCs compared with that in negative control VSMCs (Figure 1D).

As reported, VSMC migration plays an important role in the development of ASO.^6^ In our study, we found that miR‐130a significantly decreased the migration of VSMCs (Figure 1E). According to the wound closure assay, miR‐130a mimic–transfected VSMCs migrated a shorter distance than negative control cells (Figure 1F).

To sum up, these results revealed that miR‐130a suppressed VSMC proliferation and migration, which indicated the role of miR‐130a in ASO development. Meanwhile, under the consideration of the relationship between autophagy and senescence,^26^ p16 expression was detected and result showed no altered expression after miR‐130a overexpression in VSMCs (Figure S2).

### 3.2 miR‐130a inhibited VSMC autophagy

**FIGURE 2:**
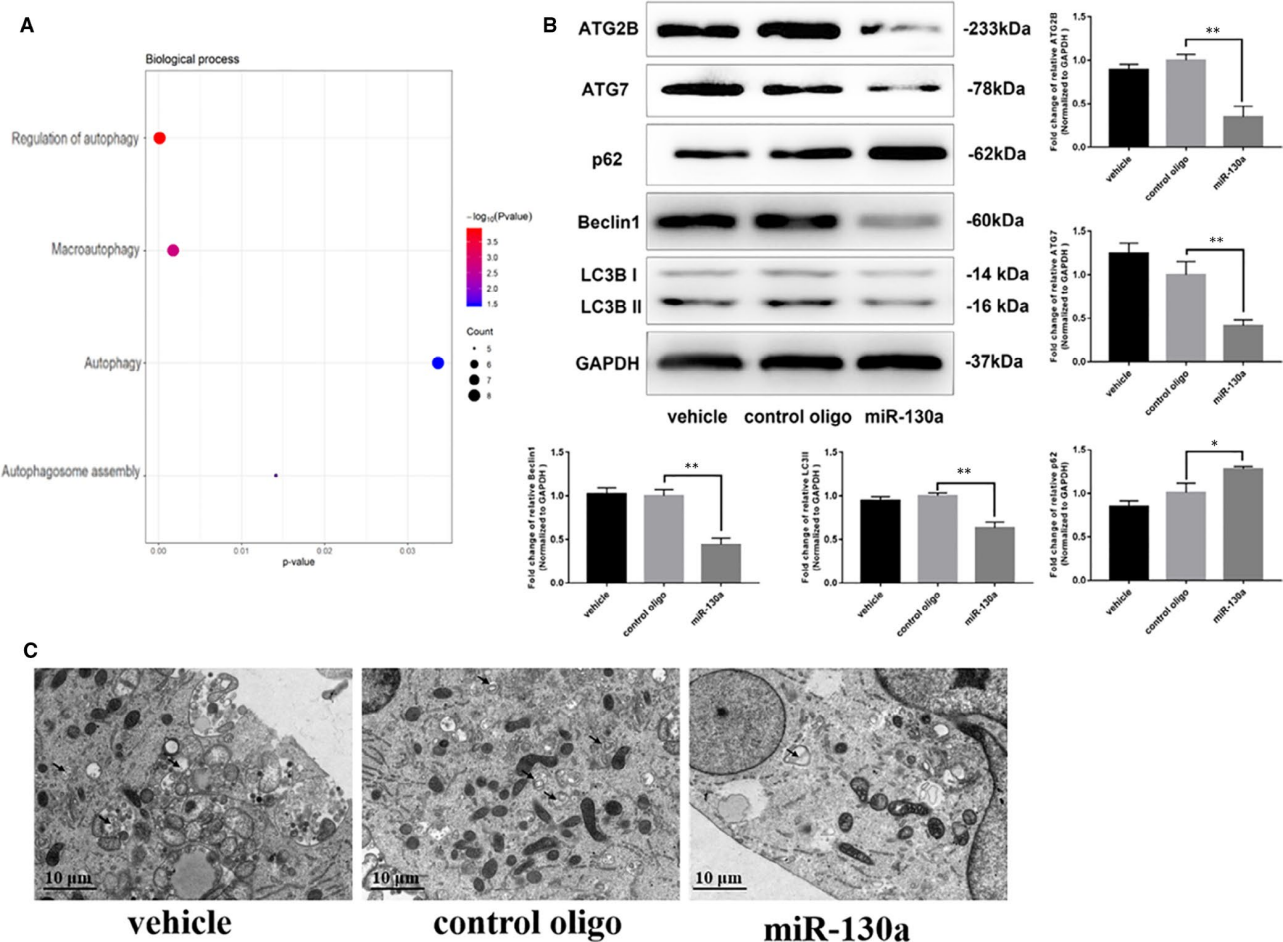
miR‐130a inhibited VSMC autophagy. A, Bubble plot of miR‐130a target genes from the GO enrichment analysis. B, The levels of ATG2B, ATG7, p62, LC3 II/I, Beclin1 and host cell GAPDH protein were measured by Western blotting as described in the Materials and Methods (n = 3 for both). C, Autophagosomes (indicated by the black arrow) were measured by TEM in each group. Scale bar = 10 μm *P < 0.05; **P < 0.005; ***P < 0.001

Based on the common gene predictions produced by the four databases (TargetScan, miRanda, Proteomics and miRmap), we identified that miR‐130a target genes were highly enriched in the GO term autophagy (Figure 2A). Previous studies revealed the role of miR‐130a in regulating autophagy and the relationship between autophagy and proliferation in ASO. Thus, we explored autophagy in VSMCs overexpressing miR‐130a. Compared with the control cells, transfection with miR‐130a mimics in VSMCs distinctly inhibited ATG7, ATG2B, LC3 II/I and Beclin1 expression and increased p62 expression (Figure 2B). Autophagosomes detected by TEM were decreased in miR‐130a–overexpressed VSMCs, which was consistent with results of Western blot (Figure 2C). These results indicated the suppression of autophagy after miR‐130a overexpression in VSMCs.

Therefore, the results above indicated that miR‐130a is closely related to autophagy according to the bioinformatic analysis and that miR‐130a could inhibit VSMC autophagy.

### 3.3 ATG2B inhibited VSMC proliferation

**FIGURE 3:**
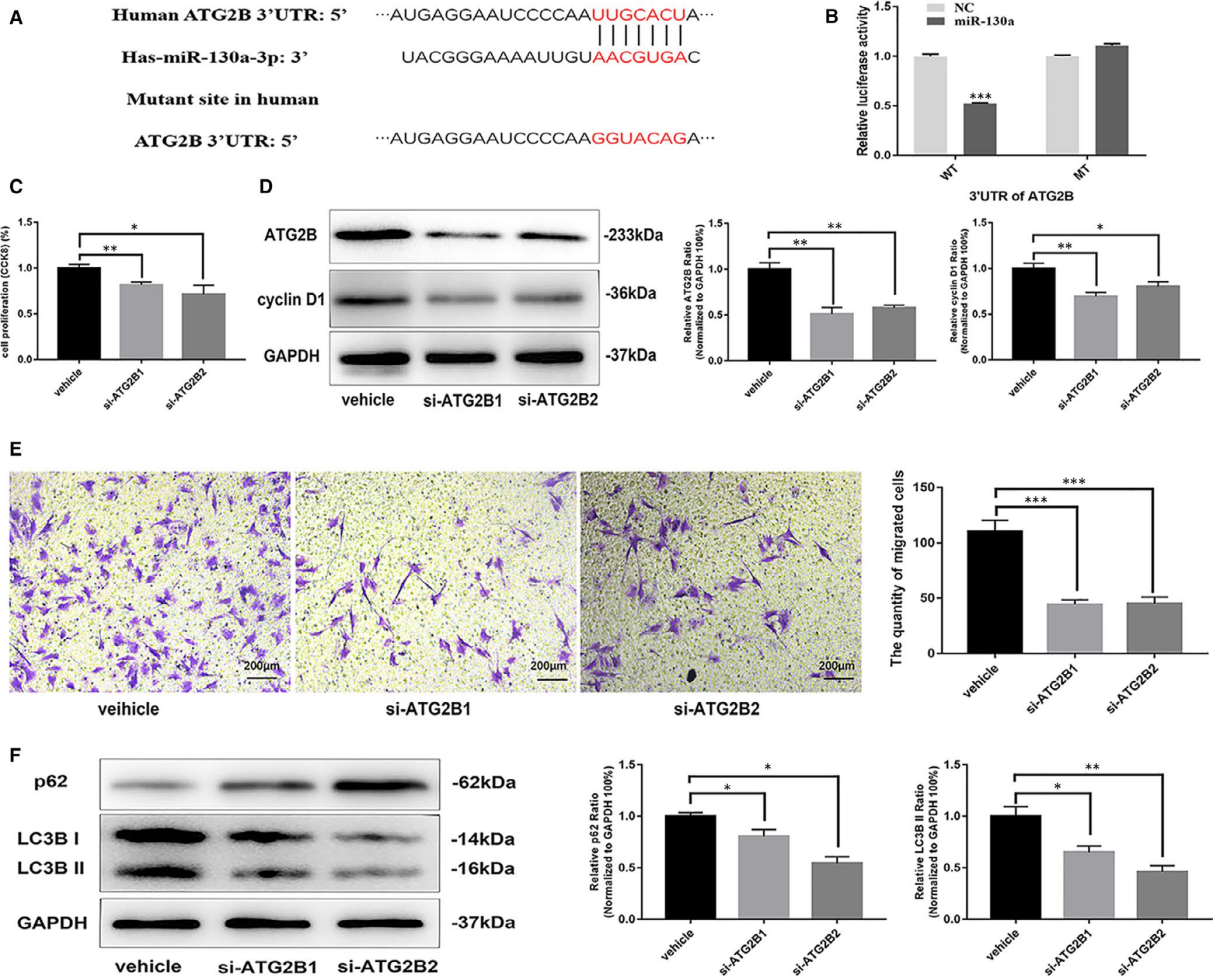
ATG2B inhibited VSMC proliferation. A, Target sequences of miR‐130a in ATG2B 3′‐UTR and mutant sites in 3′‐UTR. B, Relative luciferase activity of ATG2B 3′‐UTR and mutant in the miR‐130a mimic‐transfected 293 T cells. C, siRNA‐ATG2B decreased VSMC proliferation, as determined by CCK‐8 assay. D, The levels of ATG2B and cyclin D1 decreased when VSMCs were treated with siRNA‐ATG2B, according to Western blotting results. E, siRNA‐ATG2B decreased VSMC migration, as determined by transwell assay (n = 3 for both). Scale bar = 200 μm. F, The levels of p62, LC3 II/I and host cell GAPDH protein were measured by Western blotting. *P < 0.05; **P < 0.005; ***P < 0.001

Because miR‐130a can inhibit VSMC proliferation and autophagy, we further explored the relationship between proliferation and autophagy in VSMCs. ATG2B is one of the targets of miR‐130a according to the bioinformatic analysis. We exhibited seed sequence complementary to miR‐130a (Figure 3A). This is consistent with the literature.^23^ To confirm whether ATG2B is a direct target of miR‐130a, we performed a dual‐luciferase reporter assay. The luciferase activity was significantly reduced by miR‐130a in the presence of the ATG2B 3’UTR (wild‐type) compared with the negative control. Moreover, the inhibitory effect of miR‐130a on the luciferase activity was abrogated when we mutated the miR‐130a–binding site in the 3’UTR of ATG2B mRNA (Figure 3B). These data suggest that ATG2B is a direct target of miR‐130a. Thus, we further explored the role of ATG2B in VSMC proliferation and autophagy by knocking down its expression in VSMCs. The results demonstrated that the proliferation of VSMCs with ATG2B knockdown by transfection with two different siRNA sequences (si‐ATG2B1 and si‐ATG2B2) was significantly decreased compared with that of the negative control cells (Figure 3C). This result was validated by the decreased expression of cyclin D1 in VSMCs with ATG2B knockdown (Figure 3D). In addition, we found that knocking down ATG2B significantly decreased the migration of VSMCs (Figure 3E). Furthermore, the decreased LC3 II/I expression and increased p62 expression (Figure 3F) suggested that ATG2B knockdown inhibited VSMC autophagy. These ATG2B knockdown results were consistent with the miR‐130a overexpression results in VSMCs. Thus, the results revealed that ATG2B could inhibit proliferation in VSMCs.

### 3.4 miR‐130a inhibited proliferation by suppressing autophagy via targeting ATG2B

**FIGURE 4:**
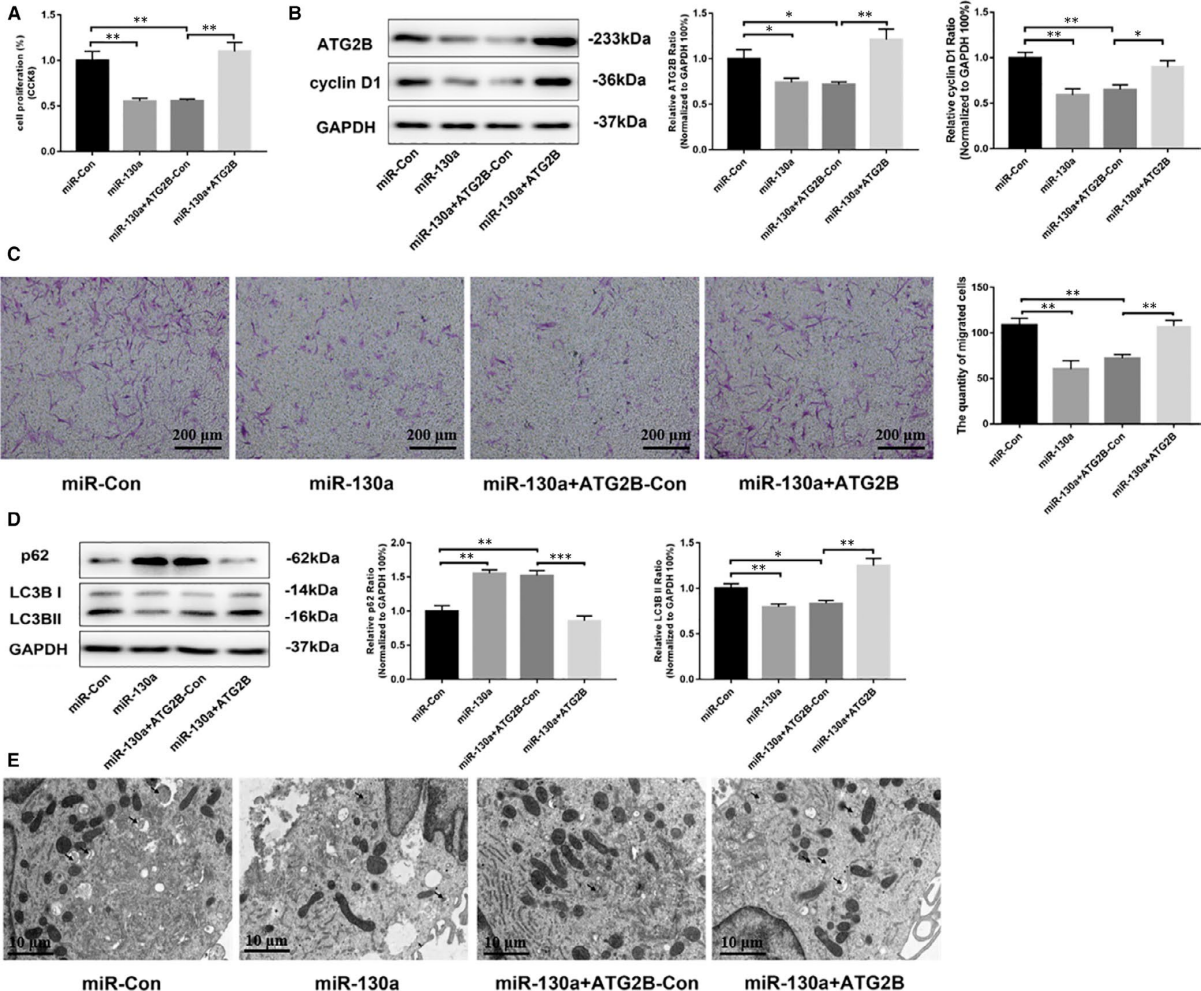
miR‐130a inhibited proliferation by suppressing autophagy via targeting ATG2B. A, ATG2B plasmid increased VSMC proliferation, as determined by CCK‐8 assay. B, The levels of cyclin D1 increased when VSMCs were treated with ATG2B plasmid, according to Western blotting results. C, ATG2B plasmid increased VSMC migration, as determined by transwell assay (n = 3 for both). Scale bar = 200 μm. D, The levels of p62, LC3 II/I and host cell GAPDH protein were measured by Western blotting. E, Autophagosomes (indicated by the black arrow) were measured by TEM in each group. Scale bar = 10 μm *P < 0.05; **P < 0.005; ***P < 0.001

To explore whether miR‐130a regulates cell proliferation and autophagy via targeting ATG2B, ATG2B plasmids and miR‐130a mimics were cotransfected in VSMCs. CCK‐8 assays showed that miR‐130a overexpression–induced growth inhibition is reversed by ATG2B plasmid transfection in VSMCs (Figure 4A). This result was validated by the increased expression of cyclin D1 in VSMCs with ATG2B overexpression (Figure 4B). In addition, we found that overexpression ATG2B significantly increased the migration of VSMCs (Figure 4C). Furthermore, the increased LC3 II/I expression and decreased p62 expression (Figure 4D) suggested that ATG2B overexpression promoted VSMC autophagy. Meanwhile, changes in autophagosomes detected by TEM were consistent with the results of Western blot (Figure 4E). Thus, the results revealed that miR‐130a could inhibit proliferation by suppressing autophagy via negatively regulating ATG2B in VSMCs.

### 3.5 miR‐130a inhibited vascular neointimal formation in balloon‐injured rat carotid arteries

**FIGURE 5:**
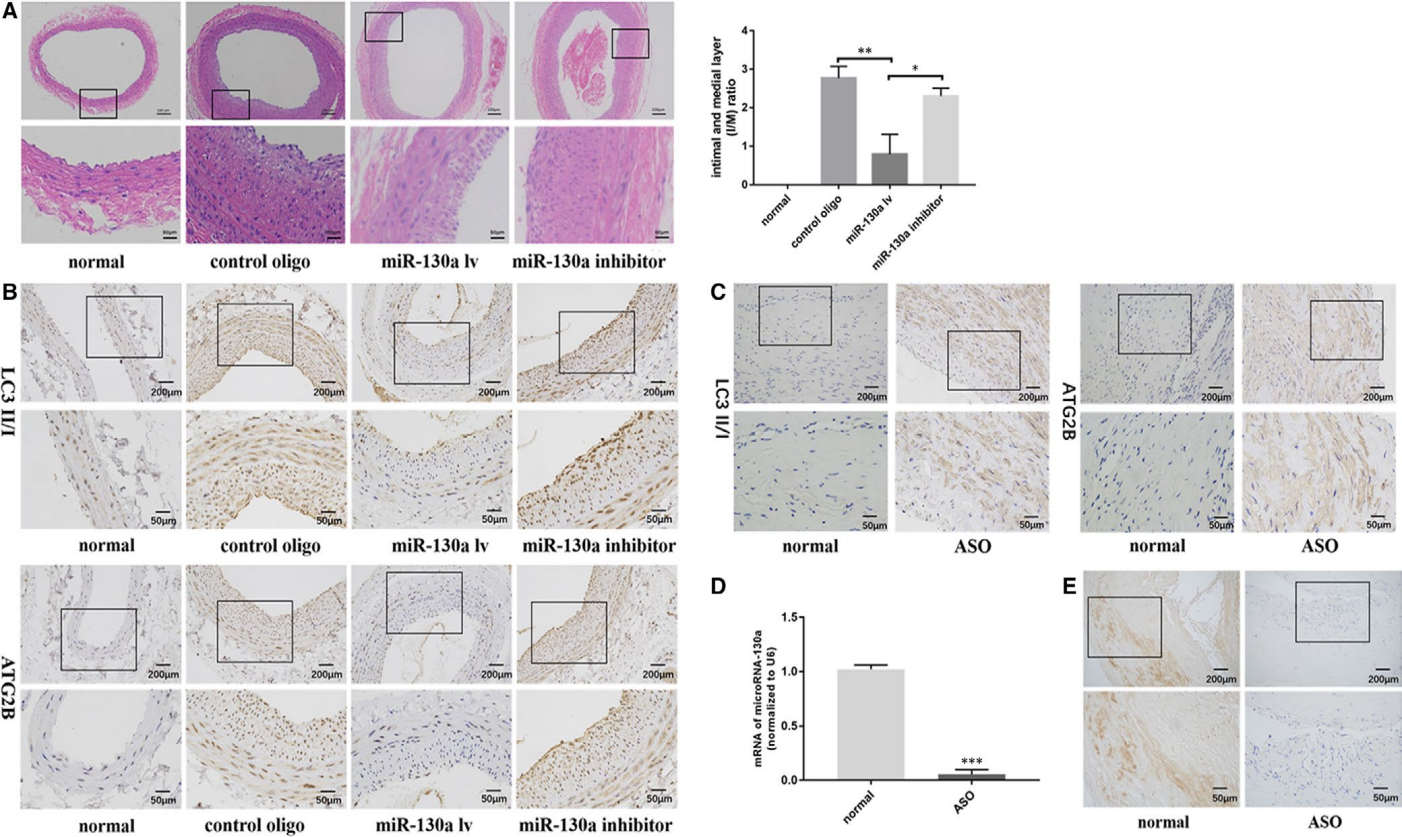
miR‐130a inhibited vascular neointimal formation in balloon‐injured rat carotid arteries. A, In the lentiviral vector‐transfected rat carotid artery balloon injury model, the degree of neointimal formation (intima‐media ratio, I/M) was decreased by the miR‐130a–overexpressing lentivirus (n = 6). Scale bar = 100 μm. B, Representative immunohistochemistry of LC3 II/I and ATG2B proteins in artery vascular tissues from rat carotid artery balloon injury model. C, Representative immunohistochemistry of LC3 II/I and ATG2B proteins in artery vascular tissues from patients with ASO or without ASO. D, qRT‐PCR analyses of miR‐130a expression levels in clinical samples. E, The expression status of miR‐130a in clinical samples detected by in situ hybridization. *P < 0.05; **P < 0.005; ***P < 0.001

We have demonstrated the role of miR‐130a in inhibiting VSMC proliferation in vitro. Thus, we examined whether miR‐130a overexpression could attenuate VSMC proliferation and neointimal formation in vivo. We used a lentivirus to deliver miR‐130a to the vascular wall after operation in the rat carotid balloon injury model.^27^ The results showed that miR‐130a overexpression significantly inhibited neointimal formation in rat carotid arteries after balloon injury compared with the control conditions, in contrast, the LV‐miR‐130a inhibitor slightly increased neointimal formation (Figure 5A). In addition, we investigated whether LC3 II/I and ATG2B were involved in miR‐130a–induced biological effects in vivo. As is shown, balloon injury significantly increased LC3 II/I and ATG2B expression, and the up‐regulation of LC3 II/I and ATG2B in balloon‐injured vascular walls was attenuated by overexpressing miR‐130a, and the situation was reversed by the LV‐miR‐130a inhibitor (Figure 5B). Furthermore, to investigate clinical significance of autophagy components (LC3 II/I and ATG2B) in ASO patients, we studied human arteries tissues with arteriosclerosis obliterans (ASO) of the lower extremities and normal lower extremity artery tissues. We detected the protein expression of LC3 II/I and ATG2B by immunohistochemistry in ASO tissues and normal tissues. We found the high expression of LC3 II/I and ATG2B in ASO patients (Figure 5C). What's more, the qRT‐PCR (Figure 5D) and the result of in situ hybridization staining (Figure 5E) are consistent with our previous research that miR‐130a expression was significantly lower in ASO arterial tissues than in control. These results from in vivo assays and clinical specimens were in accordance with in *vitro* data, and we can conclude that miR‐130a significantly attenuates VSMC proliferation and migration, which inhibits neointimal formation.

## 4 DISCUSSION

As reported, miR‐130a potentiated the metastatic behaviour of osteosarcoma cells, gastric cancer cell and cervical cancer cell.^28^, ^29^, ^30^ However, miR‐130a inhibited cell proliferation, invasion and migration in human breast cancer, chronic myeloid leukaemia cells and hepatoma cells.^31^, ^32^, ^33^ These findings suggest that miR‐130 can affect cell proliferation and migration through various pathways. In the present study, miR‐130a overexpression prevented the progression of vascular neointimal formation in balloon‐injured rat carotid arteries and inhibited cell proliferation, invasion and migration, findings that support a vascular protective role for this miRNA. The proliferation and migration of VSMCs represent key factors in the pathogenesis of ASO.^34^ He et al^35^ also reported that miR‐130a was a predictor of ASO. Our results showed that miR‐130a overexpression restricted the proliferation and migration of VSMCs. This indicated that the regulation of VSMC proliferation by miR‐130a is involved in the pathophysiological process of ASO. Thus, we further explored the mechanism by which miR‐130a regulates VSMC proliferation.

As reported, lots of studies were performed to explore the role of miR‐130a in regulating autophagy involving in various diseases. miR‐130a down‐regulation in endothelial progenitor cells (EPCs) from patients with type 2 diabetes mellitus causes dysregulated autophagy, which results in impaired EPC function.^22^ Similarly, miR‐130a was recently reported to inhibit autophagy by reducing autophagosome formation in human lymphocytic leukaemia.^23^ Additionally, miR‐130a could attenuate rapamycin/starvation‐induced autophagy in ovarian cancer cells.^21^ Lots of evidence suggests that autophagy can be activated in vascular disorders such as atherosclerosis.^36^, ^37^ However, there are no studies on miR‐130a about regulation of autophagy in VSMC. Our results indicating that miR‐130a overexpression could impair autophagy. Besides, a report revealed that VSMC treatment with the secreted protein sonic hedgehog induces autophagy in an AKT‐dependent manner and results in increased VSMC proliferation, which plays a critical role in the pathogenesis of proliferative vascular diseases such as restenosis.^15^ Furthermore, defective autophagy in VSMCs results in senescence and suppressed proliferation with G1 cell cycle arrest.^26^ However, we did not discover the altered expression of p16 in VSMCs. Thus, senescence was not found to be involved in the relationship between VSMC proliferation and autophagy by miR‐130a in our research.

Through bioinformatic analysis, ATG2B is regulated by miR‐130a. And ATG2 is a newly discovered to participate in autophagic pathways, and its ATG2B is found to be essential for autophagosome formation. And our studies have confirmed that ATG2B is a direct target of miR‐130a by luciferase assays. Meanwhile, miR‐143 targets ATG2B to inhibit autophagy and increase inflammatory reactions in Crohn's disease, and miR‐1303 also regulates mycobacteria‐induced autophagy by targeting ATG2B,^38^, ^39^ which indicated that ATG2B is the direct target of miR‐130a to regulate autophagy in VSMCs.

Furthermore, ATG2B is the direct target in the regulation that miR‐130a could down‐regulate VSMC proliferation by suppressing autophagy through in vitro assays including miR‐130a mimic overexpression, ATG2B knockdown and cotransfection with miR‐130a mimic ATG2B plasmid. Meanwhile, our results showed that miR‐130a could inhibit neointimal hyperplasia in rat carotid artery balloon injury model via autophagy inhibition, which was consistent with the results of in vitro assays.

In summary, we explored the role of miR‐130a in VSMCs and found that miR‐130a could inhibit the proliferation and migration of VSMCs both in vitro and in vivo by suppressing autophagy via ATG2B. This study implied the potential therapeutic value of miR‐130a in ASO and indicated that miR‐130a might serve as a potential target for endovascular intimal protection.

## CONFLICT OF INTEREST

All authors declare that there are no conflicts of interest.

## AUTHOR CONTRIBUTIONS

**Liang Zheng:** Data curation (lead); Writing‐original draft (lead). **Zhecun Wang:** Investigation (lead); Methodology (lead). **Zilun Li:** Formal analysis (lead); Funding acquisition (equal). **Mian Wang:** Project administration (lead); Supervision (lead). **Wenjian Wang:** Writing‐review & editing (lead). **Guangqi Chang:** Funding acquisition (equal); Writing‐review & editing (equal).

## ETHICAL APPROVAL

All animal experiments were approved by the Institutional Review Board of The First Affiliated Hospital of Sun Yat‐sen University.

## Supporting information

Fig S1

Fig S2

Supplementary Material

## Data Availability

The data sets generated and analysed during the present study are available from the corresponding author on reasonable request.
