# Supplementary figures and images for: MicroRNA‐130a inhibits proliferation of vascular smooth muscle cells by suppressing autophagy via ATG2B
Source: J Cell Mol Med. 2021 Feb 21;25(8):3829–39. doi: 10.1111/jcmm.16305 (PMC8051697; doi:10.1111/jcmm.16305)

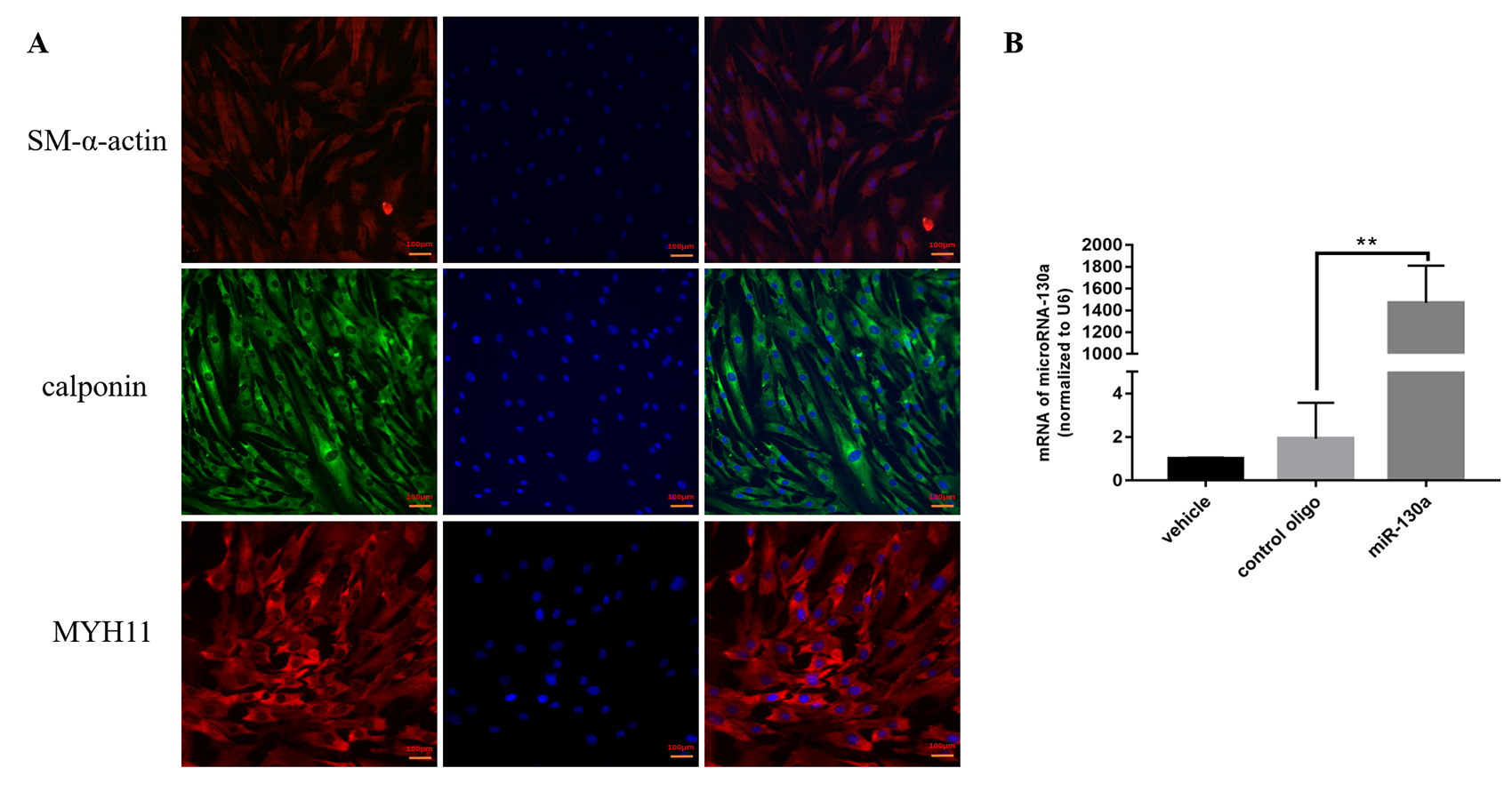

Supplement: Supplementary file 1 — Fig S1 [file JCMM-25-3829-s002.tif]

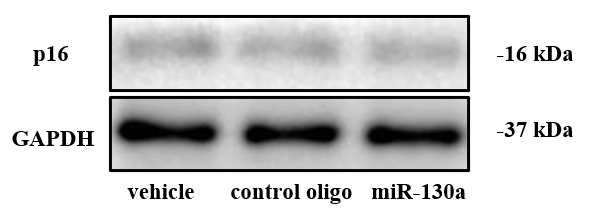

Supplement: Supplementary file 2 — Fig S2 [file JCMM-25-3829-s003.tif]
