# Supplementary material for: MicroRNA‐130a inhibits proliferation of vascular smooth muscle cells by suppressing autophagy via ATG2B
Source: J Cell Mol Med. 2021 Feb 21;25(8):3829–39. doi: 10.1111/jcmm.16305 (PMC8051697; doi:10.1111/jcmm.16305)
Supplement: Supplementary file 3 — Supplementary Material [file JCMM-25-3829-s001.docx]

**Fig. S1.** The identification of primary VSMCs. (A) Immunofluorescence of staining SM α-actin (red), calponin (green) and MYH11 (red) in VSMCs. Scale bar = 100 μm. (B) Cells were transfected with 50 nM miR-130a mimics or miR mimic negative control (MNC) with Lipofectamine RNAiMAX; mRNA level of miR-130a in VSMCs transfected with miR-130a mimics (n=3 for both). **P* < 0.05; ***P* < 0.005; ****P* < 0.001.

**Fig. S2.** The levels of p16 and host cell GAPDH were measured by western blotting. (n=3 for both).
